# The Impact of Frame Running on Quality of Life in Young Athletes With Mobility Limitations

**DOI:** 10.3389/fspor.2022.839285

**Published:** 2022-04-14

**Authors:** Petra E. M. van Schie, Mirjam van Eck, Laura A. Bonouvrié, Arnoud M. M. Edelman Bos, Annemieke I. Buizer

**Affiliations:** ^1^Amsterdam UMC Location Vrije Universiteit Amsterdam, Rehabilitation Medicine, Amsterdam, Netherlands; ^2^Amsterdam Movement Sciences, Rehabilitation and Development, Amsterdam, Netherlands; ^3^Emma Children's Hospital, Amsterdam UMC, University of Amsterdam, Amsterdam, Netherlands

**Keywords:** quality of life, psychosocial impact, adapted sports, Frame Running, children with disabilities, self-esteem, competence, participation

## Abstract

**Purpose:**

The para-athletic sport Frame Running is developed for persons with neurological impairments causing severe limitations of walking ability. Participating in sports can contribute to a better quality of life (QoL). It is unknown if participation in Frame Running contributes to QoL in children with mobility limitations. This study aims to explore the changes in QoL in children and youth who started Frame Running.

**Materials and Methods:**

We conducted a cross-sectional study amongst young Frame Running athletes with mobility limitations due to various underlying causes, aged 6–19 years, who are members of one of the Frame Running groups in the Netherlands. For 62 athletes, parents completed the Psychosocial Impact of Assistive Devices Scale (PIADS) questionnaire (subscales: competence, adaptability, and self-esteem). For six athletes, parents were interviewed to obtain more in-depth insight in the perceived changes in the QoL of their children.

**Results:**

Parents (of 58% boys, mean age 12 years 4 months; SD 3 years 3 months; 52% supported walkers) reported a significant positive change on all three subscales of the PIADS questionnaire since their children started Frame Running. Most change was experienced in the items performance, the ability to participate, happiness and self-confidence. Quotes of the parents who were interviewed elucidated these changes.

**Conclusion:**

Frame Running increased QoL in young athletes with a mobility limitation. Frame Running may therefore be advised for these children and youth to improve QoL.

## Introduction

Sports participation has numerous health benefits for typically developing children, for physical as well as psychosocial well-being (Tomporowski et al., 2011; Eime et al., 2013). Physical activity improves cardiopulmonary health, strength, flexibility and endurance, and has been related to reduce risks for cardiovascular diseases and specific cancers (Alves et al., 2016; Kubota et al., 2017). In addition, sports participation provides opportunities for social interaction, companionship and may therefore have major benefits for social and mental well-being (Street et al., 2007; Eime et al., 2010, 2013; Seippel, 2017). As such, sports participation enhances health-related quality of life (QoL) in children and adolescents (Mitchell and Barlow, 2011; Sahlin and Lexell, 2015). Health-related quality of life is a broadly defined construct evaluating the health status from the person's perspective covering physical, emotional, mental, social, and functional domains (Bullinger et al., 2002).

Children with disabilities often experience problems in participating in sports (Shields and Synnot, 2016). For youth with mobility limitations [for example due to cerebral palsy (CP)] that limit daily living activities as well as athletic endeavors, physical activity is often a challenge (Fowler et al., 2007). Children and adolescents with disabilities less often engage in physical activities and sports compared to typically developing children and adolescents (Rimmer et al., 2004; Buffart et al., 2008; Zwier et al., 2010; van Brussel et al., 2011; Carlon et al., 2013; Lankhorst et al., 2015), while they may benefit as much or even more from the influence of sports participation and physical activities on psychosocial health (Te Velde et al., 2018), as these children and adolescents may experience low levels of self-worth and quality of life due to their physical limitations and body image concerns (Rimmer et al., 2004; Sawin and Bellin, 2010). Positive self-worth, self-perceptions, self-esteem, social support and self-efficacy are acknowledged indicators of psychosocial health and quality of life (Drakouli et al., 2015). Participation in active leisure time activities is associated with better physical well-being, improved sense of self, self-esteem, social competence, emotional well-being, increased quality of life, and social well-being in children with neurodevelopmental disabilities (Shikako-Thomas et al., 2008; Dahan-Oliel et al., 2012). Moreover, participation in sports and recreational activities promotes inclusion of children with disabilities in society.

Young people with disabilities indicated that the “lack of accessible and inclusive opportunities” was the most pertinent barrier (Wright et al., 2019). Especially for children with a severe disability it is sometimes difficult to find sports they can participate in, due to their specific motor problems. Some children who are unable to walk independently can take part in wheelchair sports, but for that they need relatively good manual abilities. For children with severe impairments in both upper and lower limbs the choice for sports is much more limited. The relatively new para-athletic sport Frame Running (formerly known as RaceRunning) has been developed for persons with disabilities and high support needs. Frame Running is a form of assisted running. The running frame (with three wheels, saddle, chest plate, and steer) supports the athletes and allows them to successfully ambulate (van der Linden et al., 2021). Frame Running is intended for individuals with a motor control impairment of a cerebral nature causing a permanent and verifiable activity limitation. Since 2015, Frame Running has been implemented in the Netherlands, and getting very popular with a fast growing numbers of athletes. Apart from the athletes with neurological conditions, also many athletes with other diagnoses who are not able to walk or run without support are engaged in Frame Running.

Although it is known that sports can contribute to increase of QoL in the general population, it is unclear whether and how Frame Running contributes to QoL in children with mobility limitations. The aim of this study is to explore changes in quality of life (e.g., competence, adaptability, and self-esteem) of young athletes since they started Frame Running. Moreover, we hypothesized that the change in QoL in children who are supported walkers would be larger because they probably have less opportunities to do sports than unsupported walkers.

## Methods

### Participants

A convenience sample was recruited from national athletic sport clubs with a Frame Running group. Young Frame Running athletes were included if they met the following inclusion criteria: (a) age between 5 and 19 years; (b) a motor disability; (c) started Frame Running at least 3 months ago; (d) parents could understand the Dutch language well-enough to fill in the questionnaires. Exclusion criteria were: parents were unable to understand the questionnaire because of language problems.

### Design

A cross-sectional research design was used. Questionnaires and interviews were conducted between April 2018 and February 2019. The study was approved by the Medical Ethical Committee (METC) of Amsterdam UMC, location Vrije Universiteit in Amsterdam. The research was conducted in line with the guidelines of Good Clinical Practice (Helsinki Declaration).

### Procedure

Frame Running athletes were invited for this research project by an information leaflet via the Frame Running trainer. All parents and also adolescents older than 12 years signed for informed consent. One parent per Frame Running athlete filled in a questionnaire. A convenience sample of six parents, the first six who agreed to participate in this part of the study, were invited for a semi-structured interview.

### Outcome Measures

Demographic information about sex, age, diagnosis, means of ambulation, number of months experience with Frame Running, and hours of Frame Running training per week was investigated by means of a general questionnaire. To be able to compare non-supported and supported walkers, the Gross Motor Function Classification System (GMFCS) (Palisano et al., 1997) descriptions for means of ambulation in daily life were used for all athletes (both with CP or other diagnoses) (Towns et al., 2018). GMFCS I and II were considered non-supported walkers; III–V were considered supported walkers.

To assess QoL quantitatively, the Psychosocial Impact of Assistive Devices Scale (PIADS) was used. The PIADS consists of a brief self-report questionnaire of 26 items, designed to assess the impact of an assistive device on psychological well-being and subjective QoL of the users (Day and Jutai, 1996; Day et al., 2002; Jutai and Day, 2002; Traversoni et al., 2018). The scale has three subscales: competence (12 items), adaptability (6 items), and self-esteem (8 items). The first subscale aims to assess the perceived impact of the device on functional independence, performance and productivity. The subscale adaptability evaluates the device-related ability to participate, along with the willingness to cope with new experiences and challenges and to adapt to different settings. The subscale self-esteem collects items referring to mood, self-confidence, self-esteem, and emotional well-being (Jutai and Day, 2002; Devitt et al., 2004). Each item on all subscales is measured on a seven-point Likert scale, ranging from −3 (maximum negative impact) to + 3 (maximum positive impact). The neutral score (zero score) represents no change or no perceived impact by using the device (Devitt et al., 2004; Traversoni et al., 2018). The results of the PIADS are reported as median scores for all three subscales and each item.

The PIADS is a reliable and valid measure and it has established content validity, discriminant validity and internal validity (Day et al., 2002; Jutai and Day, 2002). It has been shown to have good construct validity, good test–retest stability (*t*-test, *p*-values 0.77–0.85) and reliability (ICC: 0.77–0.90), internal consistency (Cronbach's alpha 0.95 for PIADS total score, 0.92 for competence subscale, 0.88 for adaptability subscale and 0.87 for self-esteem subscale) (Chae and Jo, 2014) and acceptable concurrent validity (ICC: 0.77–0.83) (Traversoni et al., 2018). It has been used in research with different assistive technologies, although not yet with the running frame. The Dutch translation was made for a European research project on the effects of an incontinence device (Macaulay et al., 2007). The PIADS was translated into Dutch by the local researcher and retranslated into English by an independent professional translator. This allowed for any difficulties with the translation to be addressed, such as changes in meaning and word identification.

A convenience sample of parents of six athletes was interviewed by means of a semi-structured interview to give qualitative in-depth information about the reasons for their scores on specific items of the PIADS.

### Data Analysis

Descriptive statistics (e.g., frequency, means, standard deviation, range, and percentage) were used to describe the participants. PIADS item scores were converted to subscale scores by using the scoring sheet (Jutai and Day, 2002). To analyze if the median subscales scores were different from the neutral score (zero score, meaning no perceived impact by using the device), the one sample *t*-test was used. To analyze differences between the supported and non-supported walkers the Mann–Whitney test was used. For data analysis Statistical Package for the Social Sciences (SPSS) version 25 was used.

The six semi-structured interviews were recorded and transcribed verbatim. For this study especially the quotes about the PIADS items in which most change was experienced by the parents were reported.

## Results

From April 2018 to February 2019, all athletes of 21 Frame Running groups across the Netherlands were invited to participate by an information leaflet via their Frame Running trainer. An unknown amount of them would probably not fulfill the inclusion criteria, mostly because they were not participating in Frame Running more than 3 months and sometimes because of age. We have only included athletes who were still involved in Frame Running. In total 62 children and youth have signed up and were included and filled in the questionnaires. Most athletes were male (58%), had CP as their main diagnosis (58%) and 52% were supported walkers (GMFCS III–V). Their mean age was 12 years and mean experience with Frame Running was 13 months. There were no significant differences in sex, age, and Frame Running experience between the athletes whose parents were interviewed and the other athletes (see Table 1 for more information).

**Table 1:** Characteristics of the participating Frame Running athletes.

	**Total group (*n* = 62)**	**Interview (*n* = 6)**
**Sex**, ***n*** **(%)**		
Girls	26 (42%)	1 (17%)
Boys	36 (58%)	5 (83%)
**Age**		
Years; mean (SD)	12 yr 4 mo (3 yr 3 mo)	10 yr 5 mo (2 yr 8 mo)
Range	5 yr 7 mo−19 yr 0 mo	6 yr 9 mo−13 yr 7 mo
**Experience Frame Running**		
Months; mean (SD)	13.0 (8.1)	11.3 (4.3)
Range (months)	3–48	6–18
**Diagnosis**, ***n*** **(%)**		
Cerebral Palsy	36 (58%)	3 (50%)
Other neurological disorders	15 (24%)	2 (33%)
*Spina Bifida (3), genetic abnormality (3), TBI (2), epilepsy/ West syndrome (2), neurodegenerative disorder (2), Neurofibromatosis (1), Sturge Weber (1), HMSN (1)*		*(Spina Bifida)*
Metabolic disease	4 (6%)	
Psychomotor delay	4 (6%)	
*Down Syndrome (3); Sotos syndrome (1)*		
Orthopedic disorders	2 (3%)	1 (17%)
*AMC (1); Perthes (1)*		*(Perthes)*
Unknown	1 (2%)	
**Gross Motor Function Classification System;** ***n*** **(%)**		
GMFCS I^*^	14 (23%)	2 (33%)
GMFCS II^*^	16 (26%)	1 (17%)
GMFCS III	15 (24%)	3 (50%)
GMFCS IV	10 (16%)	
GMFCS V	7 (11%)	

*N, number; %, percentage; SD, standard deviation; yr, year; mo, months.*

* *Unsupported walkers.*

*TBI, traumatic brain injury; HMSN, hereditary motor and sensory neuropathy; AMC, arthrogryposis multiplex congenital; GMFCS, Gross Motor Function Classification System*.

### PIADS Subscale and Items Scores

The median scores on the three subscales of the PIADS for the whole group were: competence 0.83 (Q1–Q3: 0.42–1.25), adaptability 0.91 (Q1–Q3: 0.50–1.50), and self-esteem 0.55 (Q1–Q3: 0.38–1.25) meaning an increase. The median scores for all three subscales were significantly different from zero (0) (*p* < 0.001; one-sample Wilcoxon Signed Rank Test).

There were no significant differences between the non-supported walkers vs. the supported walkers in baseline characteristics (sex *p* = 0.473, age *p* = 0.293, Frame Running experience *p* = 0.841) or in median scores of the three subscales (*p* = 0.729 for competence; *p* = 0.365 for adaptability; *p* = 0.899 for self-esteem) (see Table 2).

**Table 2:** Results on PIADS domains and item scores of Frame Running athletes.

	**Unsupported walkers (*n* = 30)**	**Supported walkers (*n* = 32)**	**Total group (*n* = 62)**
	**Median (Q1 to Q3)**	**Median (Q1 to Q3)**	**Median (Q1 to Q3)**
**PIADS**
***Competence***	***0.92 (0.42 to 1.25)***	***0.75 (0.42 to 1.25)***	0.83 (0.42 to 1.25)^*^
Competence	1.00 (0.00 to 2.00)	0.00 (0.00 to 2.00)	1.00 (0.00 to 2.00)
Independence	1.00 (0.00 to 2.00)	0.00 (0.00 to 2.00)	1.00 (0.00 to 2.00)
Adequacy	0.00 (0.00 to 1.00)	0.00 (0.00 to 1.00)	0.00 (0.00 to 1.00)
Confusion	0.00 (0.00 to 0.00)	0.00 (0.00 to 0.00)	0.00 (0.00 to 0.00)
Efficiency	1.00 (0.00 to 1.00)	0.00 (0.00 to 1.00)	0.00 (0.00 to 1.00)
Productivity	1.00 (0.00 to 2.00)	1.00 (0.00 to 2.00)	1.00 (0.00 to 2.00)
Usefulness	0.00 (0.00 to 1.00)	1.00 (0.00 to 2.00)	0.00 (0.00 to 1.25)
Expertise	0.00 (0.00 to 1.00)	1.00 (0.00 to 2.00)	0.00 (0.00 to 1.00)
Skillfulness	0.00 (0.00 to 1.00)	1.00 (0.00 to 2.00)	1.00 (0.00 to 1.00)
Capability	1.00 (0.00 to 2.00)	1.00 (0.00 to 2.00)	1.00 (0.00 to 2.00)
Quality of life	2.00 (0.00 to 2.00)	1.00 (1.00 to 2.00)	1.00 (0.75 to 2.00)
Performance	2.00 (1.00 to 2.00)	2.00 (1.00 to 2.00)	2.00 (1.00 to 2.00)
***Adaptability***	***1.00 (0.67 to 1.50)***	***0.83 (0.33 to 1.33)***	0.92 (0.50 to 1.50)^*^
Well-being	1.00 (1.00 to 2.00)	2.00 (0.00 to 2.00)	1.00 (0.75 to 2.00)
Willingness to take chances	1.00 (0.00 to 1.00)	1.00 (0.00 to 1.00)	1.00 (0.00 to 1.00)
Eagerness to try new things	1.00 (0.00 to 2.00)	1.00 (0.00 to 2.00)	1.00 (0.00 to 2.00)
Ability to participate	2.00 (1.00 to 3.00)	2.00 (1.00 to 2.00)	2.00 (1.00 to 3.00)
Ability to adapt to the activities of daily living	1.00 (0.00 to 1.00)	0.00 (0.00 to 1.00)	0.00 (0.00 to 1.00)
Ability to take advantage of opportunities	1.00 (0.00 to 1.00)	0.00 (0.00 to 1.00)	0.00 (0.00 to 1.00)
***Self-esteem***	***0.75 (0.50 to 1.25)***	***0.88 (0.38 to 1.13)***	0.75 (0.38 to 1.25)^*^
Happiness	2.00 (1.00 to 2.00)	2.00 (1.00 to 2.00	2.00 (1.00 to 2.00)
Self-esteem	1.00 (1.00 to 2.00)	1.00 (0.00 to 2.00)	1.00 (0.00 to 2.00)
Security	1.00 (0.00 to 1.00)	0.00 (0.00 to 1.00)	1.00 (0.00 to 1.00)
Frustration	0.00 (−1.00 to 0.00)	0.00 (0.00 to 0.00)	0.00 (−0.25 to 0.00)
Self-confidence	2.00 (1.00 to 2.00)	1.00 (1.00 to 2.00)	1.50 (1.00 to 2.00)
Sense of power	1.00 (0.00 to 1.00)	0.00 (0.00 to 2.00)	0.00 (0.00 to 1.00)
Sense of control	1.00 (0.00 to 1.00)	1.00 (0.00 to 2.00)	1.00 (0.00 to 1.25)
Embarrassment	0.00 (0.00 to 0.00)	0.00 (0.00 to 0.00)	0.00 (0.00 to 0.00)
**Characteristics**
Age; mean (SD)	11 years 11 months (2 years 11 months)	12 years 10 months (3 years 8 months)	
Sex; boys (*n*; %)	16 (53%)	20 (63%)	
Frame Running experience; mean (SD)	12.8 months (8.67 months)	13.2 months (7.72 months)	

*PIADS, psychosocial impact of assistive devices scale.*

* *Subscale scores differed significantly from 0 (p < 0.001)*.

Most change was experienced in the items performance (subscale competence, median 2.00), the ability to participate (subscale adaptability, median 2.00), happiness (subscale self-esteem, median 2.00), and self-confidence (subscale self-esteem, -median 1.50) (see Table 2).

The parents who were interviewed elucidated these four items with the highest mean score by the following quotes. According to the Glossary of PIADS items (Day and Jutai, 1996), the item performance is described as “able to demonstrate your skills.” Many parents elucidated the increase in performance on the running frame, but also in activities in daily life in their child by the comments: “*He can walk more easily, so he walks more than before*,” “*Walking is really improving*,” “*He can walk much longer because with Frame Running you work on fitness and now he can sustain activities in daily life longer*.”

The item ability to participate is described as “ability to join in activities with other people.” It is clear that Frame Running makes it possible for athletes to participate, in the Frame Running club itself, but also in competition or running events, together with children without a disability or sometimes with their siblings or family. Moreover, the running frame can be used in daily life which makes it possible for children to participate in play. Parents elucidated this: “*He can participate in competition with Frame Running*.” One parent told: “*Especially on holidays, when we were on a camping site, she could participate and play with the other children with her running frame*.”

On the item happiness, described as “gladness, pleasure, and satisfaction with life,” all parents mentioned that their child really liked to use the running frame, it makes them happy. These are quotes: “*You can see that he really likes Frame Running, it makes him happy*.” “*She really loves to do Frame Running; she has a lot of fun, together with team mates*.” “*Every evening the day before training is fun. Then he says:* ‘*mama, tomorrow is Saturday*.' *And I ask him* ‘*what is happening then?*' *he:* ‘*I am going to do Frame Running!*”' Or this parent about the first time her daughter is trying Frame Running: ‘*If you see how much pleasure... the face of the children who were put on the running frame to try out… it is golden!*” “*He was shining the first time he was on the running frame!*”

Self-confidence, described as “self-reliance, trust in yourself, and your abilities,” of the Frame Running athletes increased as illustrated by these parents: “*She has a lot of positive experiences, playing together with other children. She is able to go out on herself, I do not have to accompany her, she feels very confident in doing it on her own*” and “*Last Sunday we participated in a 1 mile run and then she became third, surprisingly. That was a very big party for her! It has a big impact, winning something or getting a medal. It is good for her self-confidence*.” And another parent told us: “*Having success and doing things on his own makes him grow*.”

## Discussion

This study showed that parents of young Frame Running athletes reported a positive change in competence, adaptability, and self-esteem since their children started using a running frame by participating in a Frame Running athletic club. Not a single parent reported deterioration in these domains.

This is the first study, that we know of, that investigated changes in QoL since the start of Frame Running in a larger scale. The only other study which studied QoL after a period of Frame Running was the pilot study of Bryant et al. (2015). The authors introduced the Petra running frame to 15 non-ambulant children with CP in two special schools. A 12-week training period resulted in an improved ability to use the running frame. Qualitative interview data confirmed that children enjoyed using the running frame, although the authors did not find a change in data from a QoL questionnaire. This seems in line with our results of an increase in performance on the running frame, and an increase in happiness. The PIADS will be used in a recently started study on the effects of Frame Running by Ryan et al. (2020).

Our result that QoL and self-esteem increased after a period of Frame Running is in line with the findings of Te Velde et al. (2018), who showed that QoL and self-esteem was higher in children with a disability who participate in sports, in comparison with children with a disability who do not participate in sports. Maher et al. (2016) found a positive association between physical activity, social and physical quality of life, and happiness in young people with CP. Other authors reported positive effects on QoL in children with disabilities after participating in adapted soccer and swimming (Feitosa et al., 2017) and in adapted hip-hop dance practice (Withers et al., 2019). Also interventions as an adapted cycling program had a positive effect on the emotional well-being of children with CP (Demuth et al., 2012; Pickering et al., 2013a,b). Moreover, several intervention studies on fitness training in children with CP reported positive short-term effects on QoL (Verschuren et al., 2007; Demuth et al., 2012) and on social participation (Verschuren et al., 2007).

Besides the positive change in all three subscales of the PIADS, we also looked more in detail at the items. Most change was experienced in the items performance, the ability to participate, happiness and self-confidence. Our result of a rather big change in the item “ability to participate” is in line with research by Pickering et al. (2013a,b). They showed that social participation of children with CP improved when they used an adapted bicycle. Also Jeffress and Brown (2017) showed that children who played power soccer (in a wheelchair) also felt more able to participate. Using a device can improve the ability to participate in a positive way. Parents reported that their children were more often able to participate, in the sport Frame Running itself at a regular athletics club, but also in play situations at school or at home, when the child was using the running frame. Sports in general has psychosocial benefits for the participants; it gives them more independency and the feeling they can fully participate in sports.

The increase in happiness and self-confidence we have found is in line with the study of Pickering et al. (2013a,b). They concluded that the children with CP who took part in an adapted cycling program enjoyed this experience and it improved their sense of well-being. When sports is fun and makes the children happy, it is more likely that they will continue to do sports.

In our study, both non-supported and supported walkers experienced the same increase in QoL which is a positive finding. Our hypothesis that Frame Running would be more beneficial for the supported walkers was not confirmed. Although Frame Running was developed for persons with severe motor disabilities based on neurological impairments, it also seems a good option for children with less severe motor disabilities or from another than neurological origin who cannot participate in regular sports.

### Limitations

This study has some limitations. We have asked the parents to fill in the PIADS questionnaire instead of asking the athletes themselves. A recommendation for future research could be to ask the athletes to fill in the questionnaire, although for younger children and children with a cognitive disability it would probably be hard to understand the items of the PIADS. Moreover, we only interviewed parents and not the athletes themselves. In future research we recommend that young athletes themselves be included to learn about their personal experience.

There was a large range in experience with Frame Running. For the parents, recalling health status 2 years ago is very different than recalling 3 months ago. This could have affected the results by recall bias. Another limitation is a possible selection bias, because by the nature of this study, we only have data from athletes who were still using the running frame. We do not know what athletes who have stopped using the running frame would score. Also children who might have liked Frame Running, but their parents didn't (or did not have the means to support it) are not included. A recommendation for future research would be to recruit participants from the first time they tried the sport and then examine them after a fixed time frame, independent if they continued or not.

The cross-sectional design could be seen as a limitation because there is no comparison before and after introducing Frame Running or with those who are no longer active. The PIADS questionnaire measures a change in QoL, so is applicable for this design. A recommendation for future research could be to use a pre-post design with another QoL questionnaire and with a fixed time frame.

The heterogeneity of the study population could be seen as a limitation, although this is a real life representation of the Frame Running population.

## Conclusion

We found improvement in the QoL of children and youth with a mobility limitation since they started using a running frame to participate in the sport Frame Running. Our results showed that the para-athletic sport Frame Running contributes to a positive change in competence, adaptability, and self-esteem of children with a mobility limitation.

## Data Availability Statement

The raw data supporting the conclusions of this article will be made available by the authors, without undue reservation.

## Ethics Statement

The studies involving human participants were reviewed and approved by Medical Ethical Committee (METC) of Amsterdam UMC, location Vrije Universiteit in Amsterdam. Written informed consent to participate in this study was provided by the participants' legal guardian/next of kin.

## Author Contributions

PS, ME, LB, AE, and AB designed the study. PS and ME collected data, summarized results, and drafted the manuscript. All authors have reviewed and edited the manuscript.

## Conflict of Interest

The authors declare that the research was conducted in the absence of any commercial or financial relationships that could be construed as a potential conflict of interest.

## Publisher's Note

All claims expressed in this article are solely those of the authors and do not necessarily represent those of their affiliated organizations, or those of the publisher, the editors and the reviewers. Any product that may be evaluated in this article, or claim that may be made by its manufacturer, is not guaranteed or endorsed by the publisher.
