# Supplementary material for: The Impact of Frame Running on Quality of Life in Young Athletes With Mobility Limitations
Source: Front Sports Act Living. 2022 Apr 14;4:839285. doi: 10.3389/fspor.2022.839285 (PMC9046783; doi:10.3389/fspor.2022.839285)
Supplement: Supplementary file 1 [file Data_Sheet_1.pdf]

Supplement 1 – Interview guide

1. How did you get to know Frame Running?
2. Why did you/your child choose Frame Running?
3. What is your overall experience with the sport Frame Running?
    a. Positive experiences
    b. Negative experiences
4. What do you think is the impact of Frame Running on your child?
    a. On the family?
    b. On self-confidence, self-image?
5. What changes have you noticed?
    a. Positive experiences
    b. Negative experiences
6. Would you like to add something to the interview yourself?
